# Comparison of the Treatment Outcome of Piperacillin-Tazobactam versus Carbapenems for Patients with Bacteremia Caused by Extended-Spectrum β-Lactamase-Producing Escherichia coli in Areas with Low Frequency of Coproduction of OXA-1: a Preliminary Analysis

**DOI:** 10.1128/spectrum.02206-22

**Published:** 2022-08-02

**Authors:** Kosuke Hoashi, Brian Hayama, Masahiro Suzuki, Aki Sakurai, Kazumi Takehana, Taisuke Enokida, Koichi Takeda, Daisuke Ohkushi, Yohei Doi, Sohei Harada

**Affiliations:** a Department of Infectious Diseases, Cancer Institute Hospital, Japanese Foundation for Cancer Research, Tokyo, Japan; b Department of Hematology, Iizuka Hospital, Iizuka, Fukuoka, Japan; c Department of Microbiology, Fujita Health University School of Medicine, Toyoake, Aichi, Japan; d Clinical Laboratory, Cancer Institute Hospital, Japanese Foundation for Cancer Research, Tokyo, Japan; e Division of Infectious Diseases, University of Pittsburgh School of Medicine, Pittsburgh, Pennsylvania, USA; f Department of Infection Control and Prevention, The University of Tokyo Hospital, Tokyo, Japan; Johns Hopkins Hospital

**Keywords:** ESBL, *Escherichia coli*, OXA-1, carbapenems, piperacillin-tazobactam

## Abstract

Although piperacillin-tazobactam (TZP) was shown to be less effective than carbapenems in treating bacteremia due to extended-spectrum β-lactamase-producing (ESBL)-producing organisms in a randomized controlled trial, the fact that many of the causative organisms co-produced inhibitor-resistant OXA-1 along with ESBLs may have influenced the results. In this study, we compared the therapeutic effectiveness of TZP and carbapenem in treating ESBL-producing Escherichia coli bacteremia in areas with low frequency of OXA-1 co-production. Forty patients, 14 in the TZP treatment group and 26 in the carbapenem treatment group, were included in the analysis. There were no significant differences in patient background between the two groups. Urinary tract infection or cholangitis was the source of bacteremia in 26 patients (65%), and the Pitt bacteremia score was zero or one in 35 patients (87.5%). Only four (11.4%) of the 35 causative isolates available for microbiological analysis harbored *bla*_OXA-1_, and only three (8.6%) were non-susceptible to TZP. Seventeen (48.6%) isolates carried *bla*_CTX-M-27_, none of which carried other β-lactamase genes. No significant difference in the frequency of treatment failure on day 14 of bacteremia was documented between the TZP and carbapenem treatment groups in both the crude analysis and the inverse probability of treatment weighting-adjusted analysis. This study demonstrates that TZP may be a treatment option for non-severe cases of ESBL-producing E. coli bacteremia in areas with low frequency of OXA-1 co-production.

**IMPORTANCE** Although carbapenems are considered the drug of choice for severe infections caused by extended-spectrum β-lactamase-producing (ESBL)-producing organisms, other therapeutic options are being explored to avoid increasing the selective pressure for carbapenem-resistant organisms. In this study, it was suggested that piperacillin-tazobactam may be as effective as carbapenems for the treatment of mild bacteremia caused by ESBL-producing Escherichia coli in areas where OXA-1 co-production by ESBL-producing E. coli is rare. The genetic background of each regional epidemic clone differs even among multidrug-resistant bacteria classified under the same name (e.g., ESBL-producing organisms), resulting in possible differences in the efficacy of therapeutic agents. Exploration of treatment options for multidrug-resistant organisms according to local epidemiology is worthwhile from the perspective of antimicrobial stewardship.

## INTRODUCTION

Since the 2000s, infections caused by ESBL-producing *Enterobacterales* have been increasing worldwide. Although hospital-acquired ESBL-producing K. pneumoniae was dominant in the early years, community-acquired ESBL-producing E. coli infections have become more common in recent years (1). Among ESBLs produced by E. coli isolates, CTX-M enzymes, especially CTX-M-15 enzymes, are the most common worldwide (2).

Carbapenems have been the standard treatment for severe infections with ESBL-producing bacteria. However, this practice started based on small observational studies conducted in a different epidemiological setting where ESBL-producing K. pneumoniae were more common than now (3). In recent years, therapeutic agents other than carbapenems for infections caused by ESBL-producing *Enterobacterales* have been explored with the growing concern of the worldwide spread of carbapenem-resistant Gram-negative bacteria and β-lactam/β-lactamase inhibitor combinations, including piperacillin-tazobactam (TZP), are among the candidates (4). Previous observational studies have shown mixed results, with some showing TZP to be equally effective as carbapenems and others showing conflicting findings (5, 6). Although tazobactam has an *in vitro* inhibitory effect on ESBLs, experiments in animal models have shown that therapeutic effect of TZP is impaired when the inoculum is high (7, 8). The MERINO trial was a multicenter, international randomized controlled trial to investigate whether TZP was comparable to meropenem in treating bloodstream infections caused by ESBL-producing *Enterobacterales* (9). The TZP arm showed a significantly higher mortality than the meropenem arm for bacteremia caused by ceftriaxone-resistant E. coli and K. pneumoniae, 86.0% of which produced ESBL, and the study was stopped early according to the prespecified protocol. The results suggest that the therapeutic efficacy of TZP was inferior for the treatment of ESBL-producing *Enterobacterales*, but it is uncertain whether the same is true for all settings. Importantly, 67.6% of the causative isolates of the MERINO trial co-produced OXA-1, which is a narrow-spectrum β-lactamase not inhibited by tazobactam, which may have affected the therapeutic efficacy of TZP. In fact, co-production of OXA-1 was associated with resistance to TZP among ESBL-producing E. coli isolates from bacteremia cases in the UK (10). Therefore, the therapeutic efficacy of TZP for bacteremia caused by ESBL-producing *Enterobacterales* without co-production of OXA-1 is uncertain.

In this study, we aimed to compare the therapeutic effectiveness of TZP and carbapenems for bacteremia caused by ESBL-producing E. coli in situations where co-production of OXA-1 is rare.

## RESULTS

**FIG 1:**
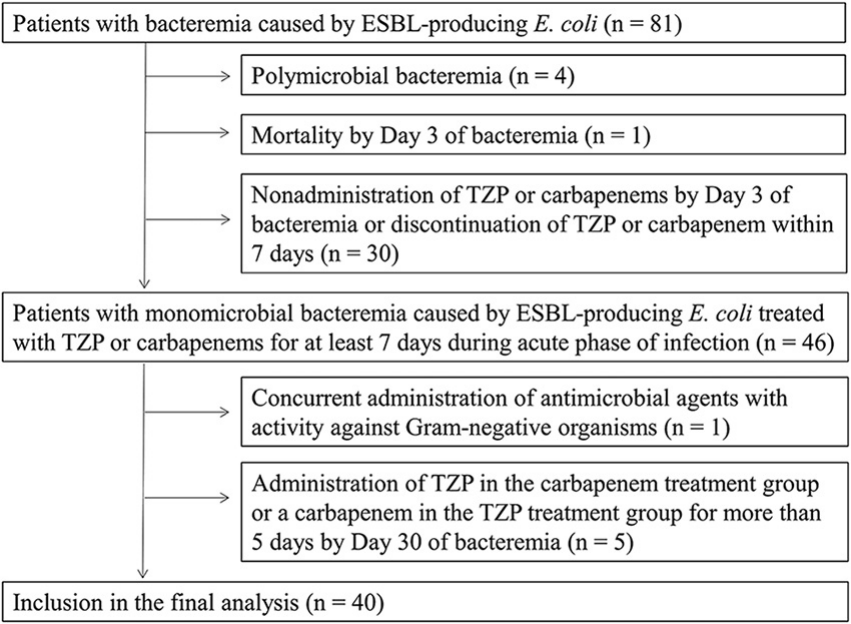
Patient inclusion. TZP, piperacillin-tazobactam.

During the study period, there were 81 cases of bacteremia caused by ESBL-producing E. coli confirmed with the disk diffusion methods at the hospital microbiology laboratory. ESBL-producing organisms in this study were defined by a positive confirmation test using the disk diffusion methods. Patients with polymicrobial bacteremia (*n* = 4), who died by day 3 of bacteremia (*n* = 1), and who were not initiated on treatment with TZP or carbapenems by day 3 or did not continue the use of TZP or carbapenems for at least 7 days (*n* = 30) were excluded from this cohort. In addition, cases in which TZP or carbapenems were administered concurrently with additional antimicrobial agents active against Gram-negative bacteria (*n* = 1) and cases in which TZP was used in the carbapenem treatment group or a carbapenem was used in the TZP treatment group for more than 5 days before day 30 (*n* = 5) were excluded. The reason for the use of TZP after the carbapenem treatment or carbapenem after the TZP treatment was drug allergy in one case and fever without documentation of ESBL-producing E. coli infection after completion of treatment for bacteremia due to ESBL-producing E. coli in four cases. As a result, 40 cases (the TZP treatment group: 14 cases, the carbapenem treatment group: 26 cases) were included in this study (Fig. 1). All cases originated from different patients. Of the 26 patients in the carbapenem treatment group, 23 were treated with meropenem, two were switched from meropenem to imipenem-cilastatin during the treatment, and the remaining one was treated with imipenem-cilastatin.

### Clinical characteristics of the patients.

Clinical characteristics of the patient in each group were demonstrated in Table 1. The mean age of the patients was 69.3 years, and 24 patients (60%) were male. Since this study was conducted at a cancer center, 37 patients (92.5%) had solid tumors, but only five patients (12.5%) had immunosuppression. Twenty-six patients (65%) had urinary-tract infections or cholangitis, which have been recognized as infections with low-risk for mortality, and no infectious complications such as disseminated infections were observed. The certainty of the source of bacteremia was definite in 25 patients (62.5%), probable in four patients (10%), possible in eight patients (20%), and unknown in three patients (7.5%). Source control for infection was achieved or unnecessary in 31 patients (77.5%) by day 3 of bacteremia. Pitt bacteremia score was zero or one in 35 patients (87.5%). Overall, no significant difference of patient background was observed between two groups.

**TABLE 1:** Clinical characteristics of the patients with bacteremia caused by ESBL-producing E. coli

Characteristic	Full cohort (*n* = 40) No (%)	Carbapenem treatment (*n* = 26) No (%)	Piperacillin-tazobactam treatment (*n* = 14) No (%)	*P* value
Age, mean y [SD: SD]	69.3 [11.2]	70.5 [10.6]	68.7 [11.6]	0.624
Age ≥ 65y	28 (70)	19 (73.1)	9 (64.3)	0.72
Male	24 (60)	17 (65.3)	7 (50)	0.5
Charlson comorbidity index ≥ 5	7 (17.5)	4 (15.4)	3 (21.4)	0.679
McCabe score				1
Nonfatal	8 (20)	5 (19.2)	3 (21.4)	
Ultimately fatal	31 (77.5)	20 (76.9)	11 (78.6)	
Rapidly fatal	1 (2.5)	1 (3.8)	0 (0)	
Solid tumor	37 (92.5)	23 (88.5)	14 (100)	0.539
Hematological malignancy	2 (5)	2 (7.7)	0 (0)	0.533
Diabetes mellitus	15 (37.5)	8 (30.8)	7 (50)	0.31
Chronic liver disease	1 (2.5)	1 (3.8)	0 (0)	1
Chronic kidney disease	1 (2.5)	1 (3.8)	0 (0)	1
Chronic heart failure	0 (0)	0 (0)	0 (0)	NA^*d*^
Immunosuppression				
Neutropenia	2 (5)	2 (7.7)	0 (0)	0.533
Cellular immunosuppression	0 (0)	0 (0)	0 (0)	NA
Humoral immunosuppression	3 (7.5)	2 (7.7)	1 (7.1)	1
Source of bacteremia				0.87
Urinary tract infection	18 (45)	11 (42.3)	7 (50)	
Biliary tract infection	8 (20)	5 (19.2)	3 (21.4)	
Intra-abdominal infection	6 (15)	4 (15.4)	2 (14.3)	
Liver abscess^*a*^	4 (10)	2 (7.7)	2 (14.3)	
Skin and soft tissue infection	1 (2.5)	1 (3.8)	0 (0)	
Unknown	3 (7.5)	3 (11.5)	0 (0)	
Setting of infection				0.609
Community-acquired	6 (15)	5 (19.2)	1 (7.1)	
Healthcare-associated	14 (35)	9 (34.6)	5 (35.7)	
Hospital-acquired	20 (50)	12 (46.2)	8 (57.1)	
Complications of bacteremia^*b*^	0 (0)	0 (0)	0 (0)	NA
Failure of source control by day 3 of bacteremia	9 (22.5)	6 (23.1)	3 (21.4)	1
ICU admission by day 3 of bacteremia	1 (2.5)	1 (3.8)	0 (0)	1
Septic shock by day 3 of bacteremia	0 (0)	0 (0)	0 (0)	NA
Pitt bacteremia score				0.3
0	14 (35)	7 (26.9)	7 (50)	
1	21 (52.5)	16 (61.5)	5 (35.7)	
2	5 (12.5)	3 (11.5)	2 (14.3)	
≥ 3	0 (0)	0 (0)	0 (0)	
Duration of treatment with the drugs of interest (carbapenems or piperacillin-tazobactam), mean days [SD]	14.5 [6.7]	15.4 [7.2]	12.7 [5.5]	0.236
Duration between the positive blood culture and initiation of the drugs of interest, mean days [SD]	1.7 [0.9]	1.9 [0.8]	1.3 [0.7]	0.022
Active antimicrobial therapy prior to the initiation of the administration of the drugs of interest^*c*^	10 (25)	10 (38.5)	0 (0)	0.007
Duration of the prior therapy, mean days [SD]	0.3 [0.6]	0.5 [0.7]	0 0	0.012
Active antimicrobial therapy after completion of the administration of the drugs of interest	22 (55)	13 (50)	9 (64.3)	0.51
Duration of the additional therapy, mean days [SD]	3.7 [4.2]	3.7 [4.6]	3.7 [3.5]	0.991

a Two cases (one case each in the carbapenem treatment group and the piperacillin-tazobactam treatment group) also had intra-abdominal infection.

b Complications include distant abscess formation, hematogenous osteomyelitis and arthritis, infective endocarditis, and septic thrombophlebitis.

c All patients with active antimicrobial therapy prior to carbapenem treatment were treated with piperacillin-tazobactam.

*^d^*NA, not applicable.

**FIG 2:**
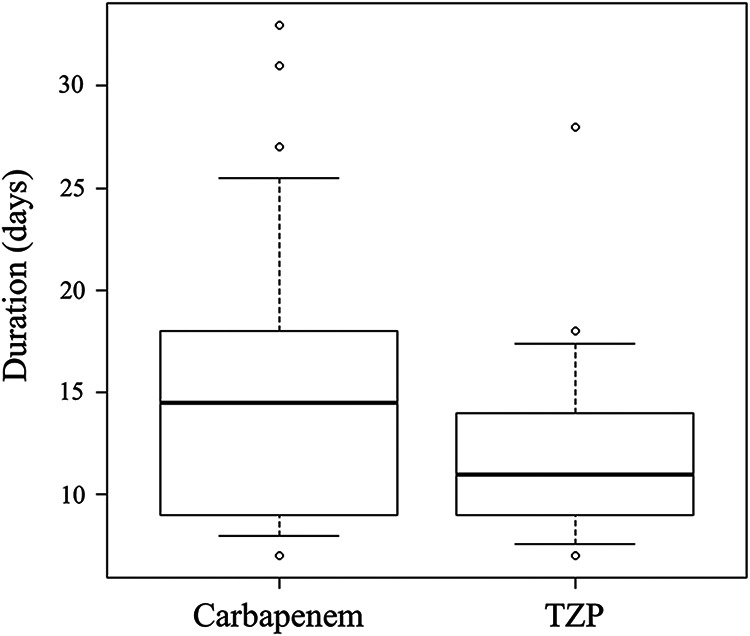
Duration of treatment with the target antimicrobial agent. Horizontal bars indicate the median, boxes indicate 25th to 75th percentile, and whiskers indicate 10th and 90th percentile. TZP, piperacillin-tazobactam.

Antimicrobial therapy with the drugs of interest (carbapenems or TZP) was administered for a median of 14.5 days in the carbapenem treatment group and 11 days in the TZP treatment group (Fig. 2). The mean duration of therapy with the drugs was numerically longer in the carbapenem treatment group (15.4 days versus 12.7 days), but the difference was not statistically significant (*P* = 0.236). Although the duration between the positive blood culture and initiation of the drug was significantly longer in the carbapenem treatment group (1.9 days versus 1.3 days, *P* = 0.022), active antimicrobial therapy prior to the initiation of the administration of the drug was also significantly longer in the carbapenem treatment group (0.5 days versus 0 days, *P* = 0.012), which appears to compensate the delay of the initiation of the analyzed drugs. Actually, all 10 patients with active antimicrobial therapy prior to carbapenem treatment were treated with TZP. More than half of all cases received additional short-term active therapy after completion of the treatment course with the analyzed drugs mostly as a form of oral step-down therapy.

### Microbiological and molecular characteristics of the causative organisms.

Among the isolates of ESBL-producing E. coli that caused the bacteremia, 35 isolates that had been frozen-stored were available for analysis (the TZP treatment group: 14 isolates, the carbapenem treatment group: 21 isolates) (Table 2, Table S1). All isolates were susceptible to imipenem and meropenem. All except three isolates in the carbapenem treatment group were susceptible to TZP. Among TZP-susceptible isolates, all except one isolate from the TZP treatment group showed MIC of ≤8/4 μg/mL against TZP. Thirty-three isolates (94.3%) were susceptible to cefmetazole, which is often used as definitive therapy for non-severe infections caused by ESBL E. coli in Japan.

**TABLE 2:** Microbiological and molecular characteristics of ESBL-producing E. coli isolates

Characteristic	Full cohort (*n* = 35) No (%)	Carbapenem treatment (*n* = 21) No (%)	Piperacillin-tazobactam treatment (*n* = 14) No (%)
Non-susceptibility to antimicrobial agents			
Amoxicillin-clavulanate	10 (28.6)	5 (23.8)	2 (14.3)
Piperacillin-tazobactam	3 (8.6)	3 (14.3)	0 (0)
Cefmetazole	2 (5.7)	1 (4.8)	1 (7.1)
Ceftibuten	19 (54.3)	14 (66.7)	5 (23.8)
Imipenem	0 (0)	0 (0)	0 (0)
Meropenem	0 (0)	0 (0)	0 (0)
Gentamicin	7 (20)	3 (14.3)	4 (28.6)
Amikacin	3 (8.6)	1 (4.8)	2 (14.3)
Ciprofloxacin	30 (85.7)	18 (85.7)	12 (85.7)
Sulfamethoxazole-trimethoprim	26 (74.3)	13 (61.9)	13 (92.9)
Fosfomycin	0 (0)	0 (0)	0 (0)
Minimum inhibitory concentration of piperacillin-tazobactam			
≤ 8/4	31 (88.6)	18 (85.7)	13 (92.9)
16/4	1 (2.9)	0 (0)	1 (7.1)
32/4-64/4	1 (2.9)	1 (4.8)	0 (0)
≥ 128/4	2 (5.7)	2 (9.5)	0 (0)
β-lactamase genes			
*bla*_CTX-M-27_	17 (48.6)	7 (33.3)	10 (71.4)
*bla*_CTX-M-14_	5 (14.3)	4 (19.0)	1 (7.1)
*bla*_CTX-M-15_	3 (8.6)	3 (14.3)	0 (0)
*bla*_CTX-M-55_	1 (2.9)	1 (4.8)	0 (0)
*bla*_CTX-M-8_	1 (2.9)	1 (4.8)	0 (0)
*bla*_CTX-M-14_^*a*^, *bla*_TEM-1B_	2 (5.7)	1 (4.8)	1 (7.1)
*bla*_CTX-M-55_, *bla*_TEM-1B_	1 (2.9)	1 (4.8)	0 (0)
*bla*_CTX-M-14_, *bla*_CTX-M-101_, *bla*_TEM-1B_	1 (2.9)	1 (4.8)	0 (0)
*bla*_CTX-M-15_, *bla*_OXA-1_	3 (8.6)	1 (4.8)	2 (14.3)
*bla*_CTX-M-15_, *bla*_OXA-1_, *bla*_CMY-2_	1 (2.9)	1 (4.8)	0 (0)
*fimH* type			
30	21 (60)	12 (57.1)	9 (64.3)
Other	14 (40)	9 (42.9)	5 (23.8)
Sequence type			
131	23 (65.7)	12 (57.1)	11 (78.6)
38	3 (8.6)	2 (9.5)	1 (7.1)
1193	2 (5.7)	1 (4.8)	1 (7.1)
Other^*b*^	7 (20)	6 (28.6)	1 (7.1)
Clade of ST131 isolates			
1	13 (37.1)	6 (28.6)	7 (50)
2	1 (2.9)	1 (4.8)	0 (0)
3	3 (8.6)	1 (4.8)	2 (14.3)
Singleton	6 (17.1)	4 (19.0)	2 (14.3)

a An isolate from piperacillin-tazobactam group had single nucleotide difference from *bla*_CTX-M-14_.

b An isolate each of ST10, ST58, ST589, ST617, ST648, ST2003 (carbapenem treatment group), and ST1148 (piperacillin-tazobactam treatment group).

All isolates carried *bla*_CTX-M_, with *bla*_CTX-M-27_ being the most common (*n* = 17, 48.6%). Eight isolates harbored other β-lactamase genes along with *bla*_CTX-M_, and four of these isolates harbored genes predicted to affect susceptibility to TZP (*bla*_OXA-1_: three isolates, *bla*_OXA-1_ and *bla*_CMY-2_: one isolate). Among these four isolates, only one isolate was resistant to TZP and another isolate showed relatively high MIC (16/4 μg/mL) against TZP. Two of the four strains with MIC of ≥16/4 μg/mL against TZP harbored β-lactamase genes other than *bla*_CTX-M_ (*bla*_OXA-1_: one isolates, *bla*_OXA-1_ and *bla*_CMY-2_: one isolate). Twenty-three isolates (65.7%) were classified as ST131 by MLST and 21 of them had *fimH30*. None of the 17 isolates with *bla*_CTX-M-27_ carried any other β-lactamase genes and 14 of these isolates were ST131/*fimH30*.

ST131 isolates and representative strains of global clones of ST131 (EC958: C2/H30Rx clone, S100EC: C1/H30R clone, S107EC and S108EC: C1-M27 clone) were classified into three clades and singletons by SNPs analysis (Table S2). Thirteen isolates were classified into clade 1 (SNPs difference: 5-112) with S107EC and S108EC (C1-M27 clone), one isolate was classified into clade 2 with S100EC (C1/H30R) with 23 SNPs difference, and three isolates carrying *bla*_CTX-M-15_ and *bla*_OXA-1_ were classified into clade 3 (SNPs difference: 63-70). No isolates were clustered with EC958 (C2/H30Rx clone). Among 14 ST131/*fimH30* isolates carrying *bla*_CTX-M-27_, 12 isolates belonged to clade 1 (Table S1). Five isolates of clade 1 had only 5–15 SNPs suggesting possible nosocomial transmission of these isolates.

### Outcomes of treatment with TZP or carbapenems.

The primary outcome, treatment failure at day 14, was observed in five patients in the carbapenem treatment group (19.2%) and two patients in the TZP treatment group (14.3%) (odds ratio [OR] 1.42 [95% CI 0.19–17.1], *P* = 1). The secondary outcome, all-cause mortality at day 30, was observed in three patients in the carbapenem treatment group (11.5%) and in none of the patients in the TZP treatment group (0%) (OR ∞ [95% CI 0.22-∞], *P* = 0.539). CDI by day 30 occurred only in one patient of the TZP treatment group.

The propensity score created for the IPTW-adjusted analysis had a c-statistics of 0.722 [95% CI 0.625–0.919]. The distribution of the propensity score between groups was balanced after adjustment with IPTW (Fig. S1). In the IPTW-adjusted cohort, the OR for the primary endpoint of the carbapenem treatment group compared with the TZP treatment group was 1.26 [95%CI 0.19–8.59] (*P* = 0.809).

Treatment outcomes were also compared only for the 35 cases for which strain was available for microbiological and molecular analysis (the TZP treatment group: 14 cases, the carbapenem treatment group: 21 cases). The primary outcome was observed in five patients in the carbapenem treatment group (23.8%) and two patients in the TZP treatment group (14.3%) (OR 1.84 [95% CI 0.25–22.5], *P* = 0.676). The secondary outcome was observed in three patients in the carbapenem treatment group (14.3%) and in none of the patients in the TZP treatment group (0%) (OR ∞ [95% CI 0.28-∞], *P* = 0.259).

Three strains with MIC of ≥16/4 μg/mL against TZP were found in the carbapenem treatment group and one strain in the TZP treatment group. Among these cases, the primary outcome was observed in one patient in the carbapenem treatment group and the secondary outcome was observed in another patient in the carbapenem treatment group. When treatment outcomes were compared only for the 31 cases in which MIC of the causative strain against TZP was ≤8/4 μg/mL (the TZP treatment group: 13 cases, the carbapenem treatment group: 18 cases), the primary outcome was observed in four patients in the carbapenem treatment group (22.2%) and two patients in the TZP treatment group (15.4%) (odds ratio [OR] 1.55 [95% CI 0.18–20.1], *P* = 1) and the secondary outcome was observed in two patients in the carbapenem treatment group (11.1%) and in none of the patients in the TZP treatment group (0%) (OR ∞ [95% CI 0.14-∞], *P* = 0.497).

None of the four cases in which the causative strain harbored *bla*_OXA-1_ (the TZP treatment group: two cases, the carbapenem treatment group: two cases) showed either the primary or the secondary outcome.

## DISCUSSION

In this study, we compared the effectiveness of carbapenems and TZP in the treatment of bacteremia caused by ESBL-producing E. coli. The primary outcome, treatment failure at day 14, was observed in two of 14 patients in the TZP group and the incidence was not significantly different from the carbapenem group. All-cause mortality by day 30 occurred in none of the patients in the TZP treatment group. Analysis of the available causative isolates demonstrated that ST131 isolates carrying *bla*_CTX-M-27_ were most common (17 isolates), only four isolates possessed *bla*_OXA-1_, and only three isolates, which were recovered from patients in the carbapenem group, were non-susceptible to TZP.

In the MERINO trial, patients who were treated with targeted therapy with TZP had a significantly higher mortality rate than those received targeted therapy with carbapenems (meropenem) in patients with bacteremia due to ESBL-producing organisms (9). Nevertheless, it is worth considering that there may be situations in which this result is not applicable.

Regarding the cases in the MERINO trial for which reanalysis of the causative isolates was possible, the carriage of *bla*_OXA-1_ together with ESBL genes was associated with an increased risk of 30-day mortality (11). In addition, reassessment of drug susceptibility by broth microdilution method showed that 6% of the causative isolates were non-susceptible to TZP, and no significant difference in mortality rates between the two treatment groups was observed after excluding these cases. While 67.6% of the isolates from the MERINO trial co-harbored *bla*_OXA-1_, only 11.4% of the isolates from the present study co-harbored *bla*_OXA-1_ and non-susceptibility to TZP was rare. Globally, CTX-M-15 producers were predominant among ESBL-producing E. coli, also accounting for 54.5% of the causative isolates in the MERINO trial (9). It is known that C2/H30-Rx, a subclone of ST131, has contributed significantly to the global spread of CTX-M-15-producing E. coli, and CTX-M-15-producing ST131 isolates often carry *bla*_CTX-M-15_ and *bla*_OXA-1_ on the same IncF plasmid (12, 13). In contrast, most of the isolates in this study were CTX-M-27-producing isolates and no concurrent carriage of additional β-lactamase genes, including *bla*_OXA-1_, was found in these isolates. A previous study from Japan also reported that only 17 of 581 ESBL-producing E. coli isolates harbored *bla*_OXA-1_ (14). In Japan, the proportion of CTX-M-27-producing isolates has been increasing in recent years, and many of these have been reported to belong to C1-M27 clade of ST131 (15). Of the 17 CTX-M-27-producing isolates in this study, 15 belonged to ST131 and SNP analysis suggested that 12 of them were C1-M27 clade isolates. It is theoretically plausible that the difference in the prevalence of clones of ESBL-producing E. coli in different countries could result in different patterns of co-possessed resistance genes and consequent difference in the efficacy of therapeutic agents. It was difficult to analyze the effect of high MIC against TZP and OXA-1 co-production on therapeutic efficacy because of the small number of relevant strains in this study.

Even when the causative isolate is identical, the efficacy of a therapeutic agent may vary depending on the severity of the infection. A meta-analysis reported that β-lactam/β-lactamase inhibitor combinations is not inferior to carbapenems in the treatment of urinary tract infections caused by ESBL-producing bacteria (16). In the current study, 65% of cases were urinary tract infections or cholangitis, which are recognized as having a low risk of developing severe disease. It is reasonable from the perspective of antimicrobial stewardship to expand the choice of therapeutic agents according to the severity of the infection.

Pathogens that have the same resistance genes but belong to different bacterial species exhibit different severity and prognosis of infections. In a global retrospective study investigating the prognosis of bacteremia caused by ESBL-producing bacteria, 30-day mortality was significantly higher for bacteremia caused by K. pneumoniae compared with that caused by E. coli (17). Most studies comparing the efficacy of treatment of infections caused by ESBL-producing bacteria, including the MERINO trial, have analyzed K. pneumoniae and E. coli as a single group (6, 9). Grouping pathogens with different properties as a single population may yield results that differ from those obtained by analyzing them as individual pathogens. In this study, we included only cases caused by ESBL-producing E. coli to improve the homogeneity of the patient population.

The fact that different antimicrobial agents were often used in a single treatment course as an empirical therapy and a targeted therapy makes evaluation of the therapeutic efficacy of a single antimicrobial agent difficult. In the MERINO trial, the drugs of interest were used for a mean of only 7.6 and 7.3 days out of the mean total treatment period of 13.7 and 13.2 days in the carbapenem treatment group and TZP treatment group, respectively (9). In the current study, only cases in which the same antimicrobial agent was continued for at least 7 days during acute phase of infection were included to accurately assess the efficacy of the therapeutic agents.

This study has several limitations. First, only a small number of cases treated with TZP were included. Due to the high mortality reported in the randomized controlled trial, few cases of bacteremia caused by ESBL-producing organisms are now treated with TZP. Therefore, we decided to analyze existing cases in a retrospective manner. In Japan, infections caused by ESBL-producing E. coli in patients without immunodeficiency are often treated with cefmetazole, which also contributed to the small number of cases included in this study relative to the overall number of infections (18, 19). Second, cases from a single institution were included, which may limit the generalizability of the results. However, since SNP analysis confirmed that most of the causative isolates were not genetically identical, the results may be applicable to other regions where the frequency of ESBL-producing E. coli co-harboring *bla*_OXA-1_ is low. Third, some cases of bacteremia due to ESBL-producing E. coli may have been missed due to false-negative results of ESBL confirmation tests by the disk diffusion method. Despite this shortcoming, we adopted this case selection criteria to reflect the situation in clinical practice.

In conclusion, carbapenems and TZP may provide similar therapeutic effectiveness against non-severe ESBL-producing E. coli bacteremia in areas where OXA-1 co-production is low. Even among organisms producing the same β-lactamase, differences in bacterial species and pattern of carriage of other resistance genes may result in different therapeutic efficacy of antimicrobial agents. Antimicrobial options should be investigated according to the characteristics of the resistant bacteria which are endemic in each region.

## MATERIALS AND METHODS

### Settings and participants.

This was a retrospective observational study performed at a 686-bed tertiary care cancer center in Tokyo, Japan, between November 2011 and September 2019. Patients with monomicrobial bacteremia caused by ESBL-producing E. coli who received TZP or carbapenems during acute phase of infection for at least seven consecutive days were selected. Identification of the bacterial species was performed with MicroScan WalkAway (Beckman Coulter, Brea, CA) and ESBL production was screened and confirmed with the disk diffusion methods as described in CLSI M100-Ed30 (20). Patients were excluded if additional antimicrobial agents active against Gram-negative bacteria were used in combination with TZP or a carbapenem, or TZP was used in the carbapenem treatment group or a carbapenem was used in the TZP treatment group for more than 5 days before day 30 of bacteremia. day 1 of bacteremia was defined as the day when a blood culture was taken from which ESBL-producing E. coli was recovered.

### Clinical data collection.

The demographic and clinical data of the patients were collected by a chart review. The following information on patient background was collected: age; sex; underlying disease such as diabetes mellitus, chronic kidney disease, chronic liver disease, neurological diseases that affect ADL, malignancy; presence of prosthetic materials in the body; McCabe index score (21); Charlson Comorbidity Index (22); immunocompromising conditions and the origins of infections (community-acquired, health care-associated, or hospital-acquired) as defined in a previous study (23). Source of bloodstream infections was determined according to the bacterial culture results of relevant clinical samples (definite), or specific radiographic or laboratory findings other than bacterial cultures (probable), or suggestive clinical signs combined with risk factors for urinary tract or biliary tract infections (possible). The severity of the infection was assessed as follows: presence of disseminated infection; ICU admission; septic shock according to sepsis-2 criteria by day 3 of bacteremia; Pitt bacteremia score (24, 25). Antimicrobial therapy by day 30 of bacteremia and source control such as drainage of abscess and removal of infected intravenous catheters were reviewed. The following outcome data were also collected: death of all cause by day 14 and day 30 of bacteremia; positive blood culture with ESBL-producing E. coli after 2 days from the initiation of TZP or carbapenem administration; improvement in symptoms related to the infection by day 7; incidence of Clostridioides difficile infection (CDI) by day 30 made by compatible symptoms and a positive toxin test by C. DIFF QUIK CHEK COMPLETE (Abbott Japan LLC, Tokyo, Japan).

### Microbiological and molecular analysis.

Isolates of ESBL-producing E. coli recovered from the eligible patients and frozen-stored according to the routine hospital protocol (the first ESBL-producing E. coli isolate from each patient was stored) were analyzed. Antimicrobial susceptibility testing was performed with the broth microdilution method according to CLSI M07-A10 (26). The results were interpreted in accordance with CLSI M100-30Ed (20). Whole genome sequencing of the isolates was performed with NextSeq 2000 (Illumina, San Diego, CA, USA). Library preparation for NextSeq 2000 sequencing was performed with QIAseq FX DNA Library kit (Qiagen, Tokyo, Japan). Libraries were sequenced on a NextSeq 2000 system for 200 cycles (100-bp paired-end reads). Raw reads generated by NextSeq 2000 were quality trimmed with fastp (version 0.23) and assembled using SPAdes (version 3.12.0). Identification of antimicrobial resistance genes, multilocus sequence typing (MLST), and *fimH* typing were performed using ResFinder-4.1, MLST-2.0, and FimTyper-1.0, respectively, which were available at the website of Center for Genomic Epidemiology (http://www.genomicepidemiology.org/).

Single nucleotide polymorphism (SNP) identification of draft genomes of sequence type (ST) 131 isolates was performed with SNIPPY (https://vicbioinformatics.com/software.snippy.shtml). The genomic sequence of strain EC958 (Accession number: GCA_000285655) was used as the reference of ST131 isolates. The genomic sequences of S100EC (GCA_01247299), S107EC (GCA_012473595), and S108EC (GCA_012474135) were added in the analysis as the representative strains of C1/H30R clone (S100EC) and C1-M27 clone (S107EC and S108EC).

### Statistical analysis.

Continuous data are expressed as mean with standard deviations and categorical variables are shown as proportions. Clinical variables between groups of patients were compared using Fisher’s exact test and Student's *t* test for categorical variables and quantitative variables, respectively.

The primary outcome was failure of the treatment on day 14 of bacteremia defined as (1) death of all cause by day 14, (2) positive blood culture with ESBL-producing E. coli after 2 days from the initiation of TZP or carbapenem administration, or (3) no improvement in symptoms related to the infection, including fever (≥ 37.5°C) by day 7. The secondary outcomes were all-cause mortality by day 30. Outcomes were compared between patients treated with TZP and those treated with carbapenems. For the comparison of the primary outcome between groups, adjustment for confounding by indication was performed using inverse probability of treatment weighting (IPTW) in addition to the crude analysis. Age, sex, Charlson comorbidity index > 5, McCabe score, solid tumor, hematological malignancy, diabetes mellitus, chronic liver disease, chronic kidney disease, neutropenia, humoral immunosuppression, source of bacteremia, Pitt bacteremia score were included in the model to construct the propensity score for decision between the use of carbapenem and TZP. The primary outcome was compared between treatment groups adjusting for potential confounding by indication through stabilized average treatment effect (ATE).

A *P*-value < 0.05 was considered as significant for all statistical analysis. EZR (ver. 1.54) and R statistical software package (ver. 4.0.3) were used for the statistical analysis (27).

### Ethics.

Institutional review board approval was obtained before initiating the study (approval number: 2019-1188). All procedures were in accordance with the 1964 Declaration of Helsinki and its later amendments or comparable ethical standards. Informed consent for this retrospective, noninvasive study was not required by the local ethics committee.

### Data availability.

All genome sequences have been deposited in the NCBI database under BioProject accession number PRJNA836713.
