# Supplementary figures and images for: Comparison of the Treatment Outcome of Piperacillin-Tazobactam versus Carbapenems for Patients with Bacteremia Caused by Extended-Spectrum β-Lactamase-Producing Escherichia coli in Areas with Low Frequency of Coproduction of OXA-1: a Preliminary Analysis
Source: Microbiol Spectr. 2022 Aug 2;10(4):e02206-22. doi: 10.1128/spectrum.02206-22 (PMC9430612; doi:10.1128/spectrum.02206-22)

**Figure S1. Distribution of propensity score in each group before and after IPTW adjustment**

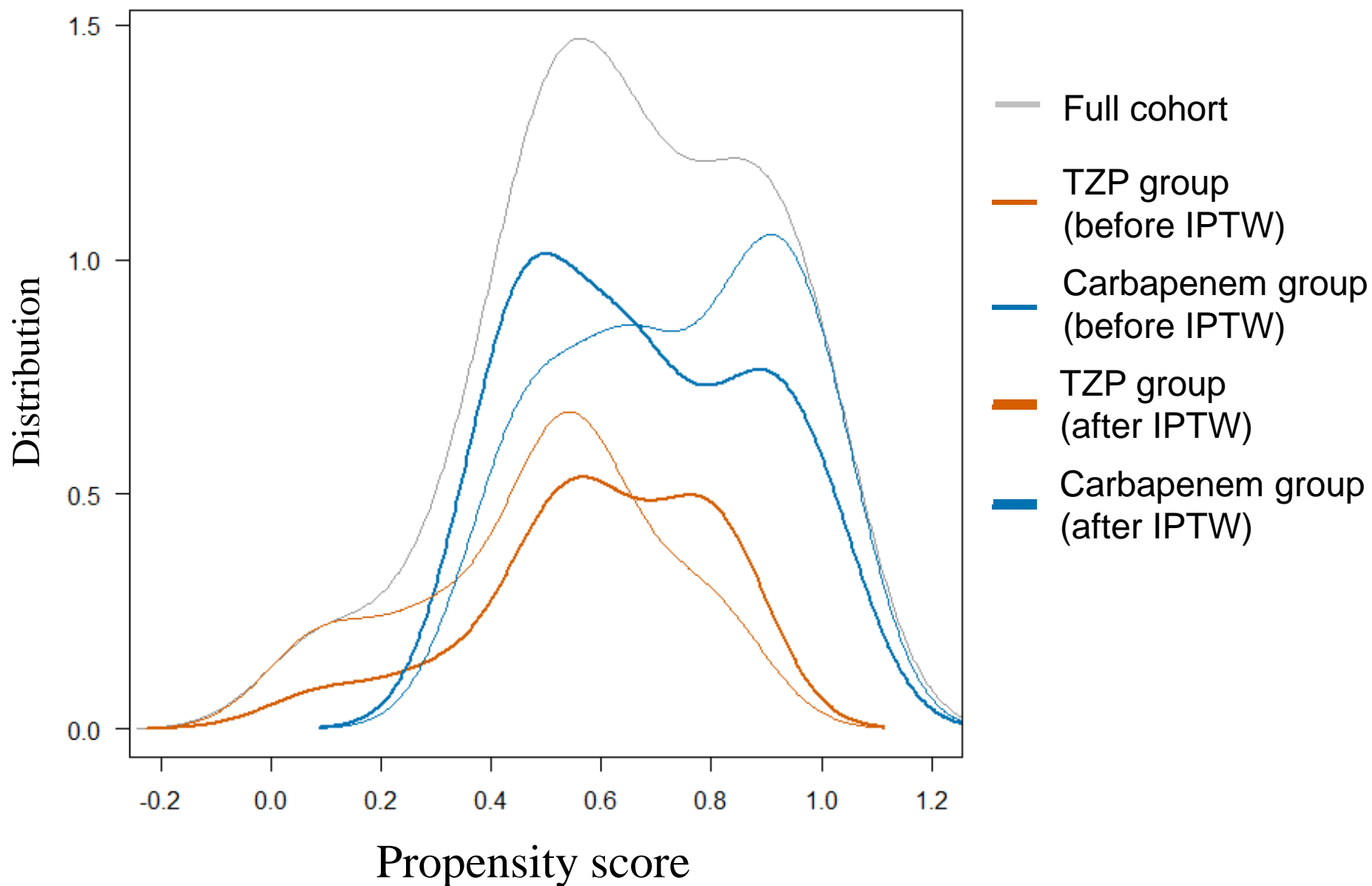

Supplement: Supplemental file 1 — Fig. S1. Download spectrum.02206-22-s0001.pdf, PDF file, 0.02 MB [file spectrum.02206-22-s0001.pdf]
